# Nutritional Status in Pediatric Psoriasis: A Case–Control Study in a Tertiary Care Referral Centre

**DOI:** 10.3390/children11070885

**Published:** 2024-07-22

**Authors:** Adelina-Maria Sendrea, Sinziana Cristea, Carmen Maria Salavastru

**Affiliations:** 1 Pediatric Dermatology Department, Carol Davila University of Medicine and Pharmacy, 8 Eroilor Sanitari Boulevard, 050474 Bucharest, Romania; carmen.salavastru@umfcd.ro; 2 Pediatric Dermatology Department, Colentina Clinical Hospital, 19-21 Stefan cel Mare Street, 020125 Bucharest, Romania; 3 Dermatology Research Unit, Colentina Clinical Hospital, 19-21 Stefan cel Mare Street, 020125 Bucharest, Romania; 4 Certara Inc., Radnor Corporate Centre, Suite 350, Radnor, PA 19087, USA; sinziana.cristea@certara.com

**Keywords:** psoriasis, pediatric PASI, BMI, obesity, overweight, NAPSI

## Abstract

Background: Psoriasis and obesity are chronic, inflammatory diseases, sharing certain pathophysiological factors. Psoriasis, increasingly viewed as a systemic inflammatory condition, may have various symptoms beyond the skin manifestations. Methods: This research aimed to explore the connection between body mass index (BMI) and pediatric psoriasis, through a case–control study on 100 psoriasis cases and 100 controls who were matched in terms of age and sex. The percentiles of the BMI by age and sex determined the nutritional status of each patient and control. The severity of psoriasis was evaluated based on the psoriasis area and severity index (PASI), nail involvement based on the nail psoriasis severity index (NAPSI), and quality of life impairment with the dermatology life quality index (DLQI). Results: While no statistically significant relationship was identified between increased BMI and PASI (*p* = 0.074), the risk of being overweight and obesity was significantly higher in the psoriasis group (OR 6.93, *p* = 0.003; OR 12.6, *p* < 0.001, respectively). The BMI increased with the PASI for psoriasis vulgaris but not for psoriasis inverse. No connections were found between disease duration and BMI (*p* = 0.56) or between BMI and PASI based on sex (*p* = 0.26). The NAPSI increased significantly with increased BMI (*p* = 0.000015). Conclusions: This study highlights the association between elevated BMI, psoriasis diagnosis, and severity of psoriatic onychopathy in pediatric patients, advocating for further large-scale studies to confirm these explorations and increasing awareness for better screening and management of such cases for overweight/obese patients.

## 1. Introduction

Psoriasis represents an inflammatory, papulosquamous chronic dermatological disorder, presenting specific cutaneous and nail lesions. Psoriasis affects up to 3.5% of the worldwide population, of which up to one-third of cases are with a childhood onset [1]. Its pathogenesis is not fully understood, relying on a complex interplay between multiple key-features, such as T helper 1 (Th1)-driven inflammation, immune disturbances, genetic background, and abnormal keratinocyte proliferation and differentiation [2]. Clinically, it presents with various types (i.e., vulgaris, guttate, inverse, erythrodermic, pustular, follicular) and severity degrees (according to the psoriasis area and severity index—PASI).

Over the past few decades, the systemic nature of psoriasis has been increasingly brought to light, focusing on the association of cardiovascular and metabolic diseases (e.g., obesity, arterial hypertension, diabetes mellitus, dyslipidemia, or metabolic syndrome), mainly in adult patients [3,4,5]. However, recent data have emphasized an increased prevalence of overweight or obesity in children with psoriasis [6], with conflicting results. While some studies identified an association between the overweight and obesity status in children with psoriasis (even directly proportional with psoriasis severity [7]), others failed to reveal any association between these two diseases [8]. Furthermore, despite some studies identifying an increased risk between psoriasis and an increased body mass index (BMI), no connection to psoriasis severity was found [9]. The potential association between these two diseases could be multifactorial, as psoriasis and obesity share various pathophysiological features—genetic background, chronic inflammation or gut microbiota disturbances [10,11,12]. Moreover, the concept of “psoriatic march” was born in the past decades in order to elucidate the connection between inflammation and cardiovascular disease in psoriasis patients. This particular concept supports the idea that, in such patients, the interplay between the perpetual, low-grade inflammation, obesity, and certain metabolic disturbances contributes to insulin resistance and endothelial dysfunction, further leading to atherosclerosis and, eventually, cardiovascular disease [13,14,15]. However, the relationship between psoriasis and obesity is not fully understood in children and remains a topic for research and debate in the literature.

Our research aimed to evaluate the potential relationship between increased BMI, indicative of overweight or obese status, and various characteristics of pediatric psoriasis, severity or clinical type, disease duration, nail involvement or quality of life impairment.

## 2. Materials and Methods

A retrospective, case–control study was conducted on 100 children diagnosed with psoriasis and 100 healthy controls having the same sex and age. All the patients included in this study presented to a tertiary care referral center from Bucharest, Romania—Pediatric Dermatology Department of Colentina Clinical Hospital—during a period of a year and a half (June 2022–December 2023).

In the case study group (psoriasis), children < 18 years old with a psoriasis diagnosis were included, while in the controls study group (i.e., non-psoriasis), the same number of children diagnosed with skin infections or infestations, with identical sex and age, were included. For both groups, patients having any documented disease (especially components of metabolic syndrome or endocrinopathies) or being previously treated with topical steroids or any systemic treatment were excluded.

Psoriasis diagnosis was established through a thorough physical examination by dermatologists with pediatric expertise. The clinical types identified were vulgaris, inverse, and guttate, and disease severity was evaluated using the PASI score (psoriasis area and severity index), cases being subdivided as mild (PASI 0–5), moderate (PASI 6–10), and severe (PASI > 11). Nail involvement was evaluated using the nail psoriasis severity index (NAPSI), and the quality-of-life impairment was appraised using the validated dermatology life quality index (DLQI) questionnaire for children aged >16 years old and its children version (children’s DLQI—CDLQI) for children aged <16 years old, respectively [16].

Demographic data (age and sex) were recorded, and patients were subdivided into the following age categories: toddler (1–2 years old), early childhood (2–5 years old), middle childhood (6–11 years old), and adolescence (12–17 years old). Additionally, anthropometric parameters (weight in kilograms and height in meters) were assessed in each research subject. The body mass index (BMI) was computed with the subsequent equation:

$$BMI\left(\frac{kg}{m^{2}}\right)=\frac{weight\left(kg\right)}{height^{2}\left(m^{2}\right)}$$

The Centers for Disease Control (CDC) BMI Calculator for Child and Teen [17] and the Ped(z) Pediatric Calculator with CDC/ World Health Organization (WHO) data [18] were used to determine the BMI and corresponding percentiles (pctl) for subjects aged beyond 2 and 2 and under, respectively. Furthermore, we assessed the nutritional status of each participant based on the following percentiles: as underweight (<5th percentile), healthy weight (5th–85th percentile), overweight (85th–<95th percentile), and obese (>95th percentile).

The descriptive statistics data for both groups included the mean, with standard deviation (SD), the median value, and the complete range of data. As the two groups included in the study were already paired exactly based on their sex and age, no comparison of these parameters between cases and controls was necessary. A paired t-test was employed to evaluate the possible connection between psoriasis and BMI and NAPSI score and BMI, respectively, using the accurate age–sex matching. A logistic regression analysis was used to calculate the odds ratio (OR) and 95% confidence intervals (CI), and the statistical significance threshold was set at a *p*-value of <0.05. The objectives of the study were to determine if (1) children with psoriasis have a greater likelihood of being either overweight or obese, compared to controls group; (2) children with greater severity grades of psoriasis have a more increased risk of being either overweight or obese, compared to those with milder disease; and (3) the likelihood that an increased BMI is directly linked to nail involvement, as evaluated through the NAPSI score. The analysis of the data and statistical tests were performed using RStudio version 2023.03.0 Build 386.

## 3. Results

### 3.1. Study Groups Characteristics

Considering that the study groups were an exact match in age and gender, no statistically significant differences were found for these demographic characteristics (*p* > 0.99) between the two groups. The mean (SD) age was 10.2 (±4.43) years (youngest patient, 1 year old and oldest, 17 years old), with the most representative age group being middle childhood (41%), followed by adolescence (40%), early childhood (18%), and toddler (1%). The gender distribution showed a slight male predominance (53%) compared to females (47%) (Table 1). No statistically significant difference between the characteristics was identified between the cases and controls groups, except for the nutritional status, where more patients in the cases were classified as overweight and obese, as compared to the controls. (*p* < 0.05) Nail involvement is only present in the cases group; hence, no *p*-value is provided.

In the controls group, the most common diagnosis was warts (48%), followed by various types of tinea (capitis—10%, corporis—12%, cruris—1%, pedis—1%), scabies (9%), impetigo (8%), herpes simplex infection and herpes zoster (each 4%), and, finally, pediculosis capitis and molluscum contagiosum (each 1%). In the psoriasis group, the most common type was vulgaris (62%), followed by inverse (31%) and guttate (7%).

The BMI was comparable between the psoriasis cases group and controls (19.4 vs. 18.4) (*p* = 0.68). However, the variability of the BMI is higher in the psoriasis cases group (SD = 5.52), as compared to the controls (SD = 3.95), which indicates a more expanded BMI interval in psoriasis patients. This finding is further supported by the increased rates of overweight status (14%) and obesity (17%) in psoriasis patients, as compared to the controls (overweight 3%, obese 2%) (*p* < 0.001). Nonetheless, the normal weight group was predominant for psoriasis (64%) and controls (95%) (Table 1).

### 3.2. BMI-Psoriasis vs. Controls

**Figure 1:**
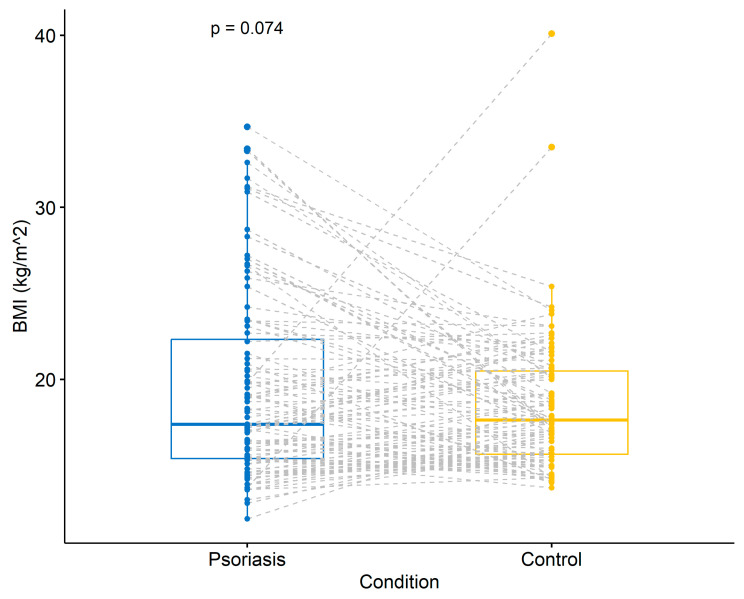
Boxplots comparing the BMI of both study groups (psoriasis (blue) vs. controls (yellow)) by performing a paired t-test. The boxplots represent the median and interquartile range (IQR). Each individual point shows the BMI measurements for each study group. The dotted gray lines match each psoriasis case with its exact control; BMI = body mass index; *p* = *p*-value.

Figure 1 shows the results of the paired t-test between the psoriasis group and their matching controls. The t-test failed to show a statistically significant disparity amongst the BMI means (*p* = 0.074) of the cases (psoriasis) group, compared to the controls (non-psoriasis) (Figure 1).

However, as the exploration of the data showed that, in the psoriasis cases group, the proportion of overweight and obese patients was higher, as compared to the controls (Table 1), we quantified a 6-fold-increased relative risk of overweight status (OR 6.93 [CI 2.16–30.9], *p* = 0.003) and a 12-fold-increased relative risk of being obese (OR 12.6 [CI 3.46–81.3], *p* < 0.001) among psoriasis patients, as compared to their matched controls. Although the 95% CI for the OR are broad, indicative of a reduced level of accuracy, none contain the null value of OR = 1.

### 3.3. Association between BMI, PASI, Psoriasis Duration, and DLQI

Based on the PASI, most psoriasis cases were evaluated as mild (65%), followed by moderate (25%) and severe (10%). The mean (SD) PASI value for psoriasis cases was 4.75 (±5.01). As expected, the PASI increases with disease severity from 2.18 in the mild group to 16.1 in the severe group (*p* < 0.001). The mean (SD) age was comparable amongst all severity types (*p* = 0.41). Even though male patients were more prevalent in the severe and mild cases subgroup (>60% males), as compared to the mild psoriasis cases subgroup, where female patients were slightly more prevalent (>50% females), the difference in distribution of gender between the 3 subgroups was similar (*p* = 0.18). The proportion of patients with overweight status and obesity grew with disease severity in each subgroup (mild psoriasis (29.2%), moderate psoriasis (32%), severe psoriasis (40%)); however, this difference was not found to be statistically significantly different (*p* = 0.79) (Table 2).

**Figure 2:**
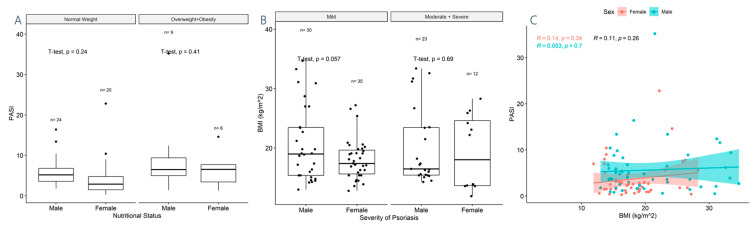
(**A**) Boxplots comparing the PASI of males and females by nutritional status by performing a paired t-test. Boxplots represent the median and interquartile range (IQR). (**B**) Boxplots comparing the BMI of males and females by psoriasis severity by performing a paired t-test. Boxplots represent the median and interquartile range (IQR) (**C**) Linear regression between BMI and PASI by gender for the psoriasis cases group. Dots show individual data. Colors correspond to each gender: pink—females; blue—males. Shaded area shows the 95% confidence interval for each gender. BMI = body mass index; PASI = psoriasis area and severity index.

A pairwise comparison of the multivariate data indicated that in the overweight and obese psoriasis cases group, the males had a higher BMI than the females (Figure S1). Furthermore, the PASI seemed to be higher in males, as compared to females in all disease severity groups (Figure S1). Therefore, these relationships were further tested for their statistical significance; however, none of these associations were found to be statistically significant following a t-test: there is no difference in the BMI between males and females neither in the mild psoriasis (*p* = 0.057) nor in the combined moderate and severe psoriasis (*p* = 0.69) groups; there is no difference in the PASI between males and females neither in the normal weight group (*p* = 0.24) nor in the combined overweight and obese group (*p* = 0.41) In Figure 2, we show that even though the PASI seems to be higher in males, as compared to females with the same BMI, there is no statistically significant association between BMI and PASI for neither males nor females. The slopes of the linear regression are similar between the two genders, with 95% CI overlapping across the entire range of data.

**Figure 3:**
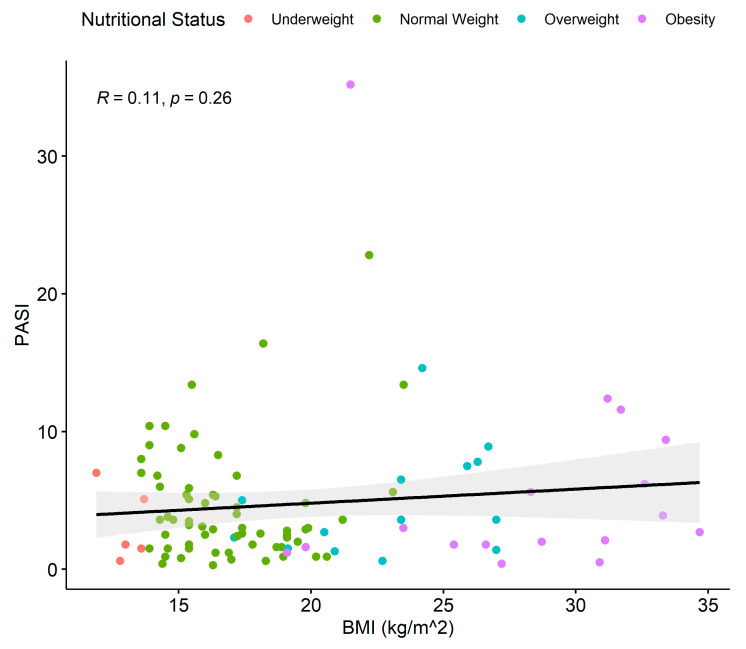
Linear regression between BMI and PASI score for the psoriasis cases group. Dots show the individual data. Colors show the nutritional status of the patients. Black line = regression line; gray area = 95% confidence interval. BMI = body mass index; PASI = psoriasis area and severity index.

Figure 3 shows the association between the BMI and PASI across the entire psoriasis cases group. However, no statistically significant association was found between elevated BMI and the severity of psoriasis, as assessed through PASI (linear regression, *p* = 0.26) (Figure 3). This is further confirmed by the OR, as the relative risk of the association of overweight status or obesity in moderate–severe psoriasis, compared to a mild one remained statistically insignificant (OR 1.29 [95% CI 0.52–3.15, *p* = 0.6] and 95% CI including the null value of OR = 1).

**Figure 4:**
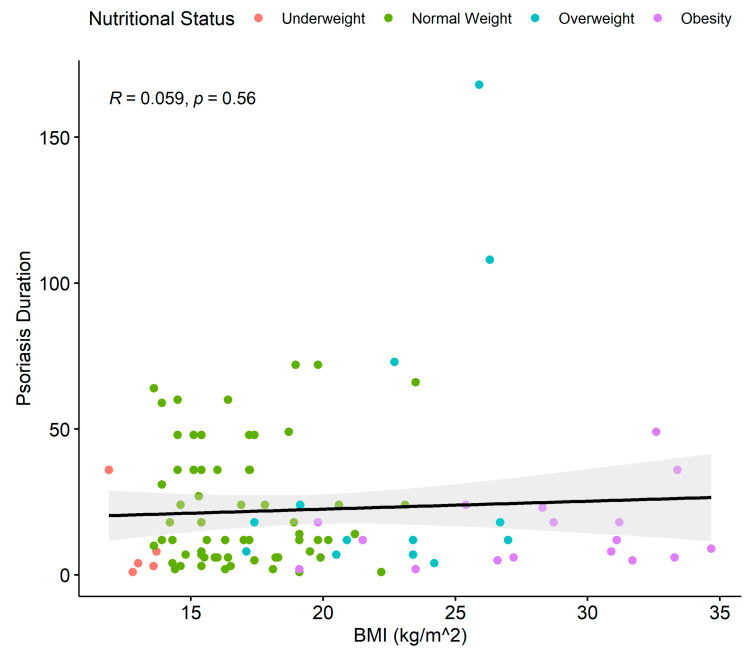
Linear regression between BMI and psoriasis disease duration (in months) for psoriasis patients. Dots show the individual data. Colors show the nutritional status of the patients: red (underweight), green (normal weight), blue (overweight), purple (obese). Black line = regression line; gray area is the 95% confidence interval; BMI = body mass index.

Additionally, we explored a potential link between the BMI and psoriasis disease duration. Following a linear regression analysis, BMI does not appear to increase with an increase in disease duration (slope = 0.01289) (*p* = 0.56) (Figure 4).

**Figure 5:**
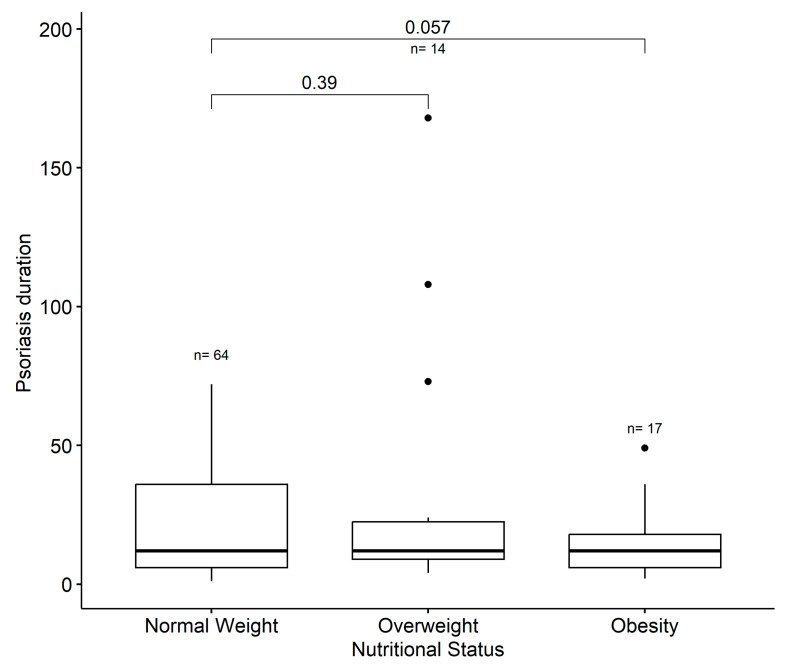
Boxplots showing the distribution of psoriasis disease duration (i.e., median with interquartile range) between three groups of nutritional status (normal weight, overweight, obesity; underweight individuals were excluded). The number of participants in each group is shown above each boxplot. The *p*-values following a t-test comparing the psoriasis duration in the normal weight group with the overweight and obese groups separately.

However, when comparing the duration of psoriasis between the nutritional status groups (with normal weight as a reference and overweight and obese as test groups), the *t*-test indicated no statistically significant difference between normal weight and overweight. Nonetheless, the analysis nearly reached the significance threshold (*p* = 0.057), indicative of a higher chance of being obese with increased duration of disease (Figure 5).

### 3.4. BMI–Psoriasis Types Association

**Figure 6:**
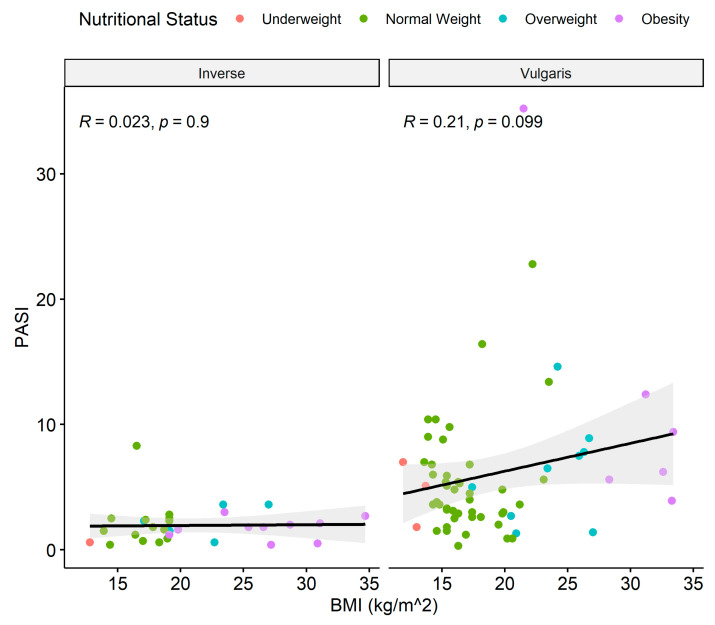
Linear regression between BMI and PASI score for psoriasis type inverse and type vulgaris. Dots show the individual data. Colors show the nutritional status of the patients. Black line = regression line; gray area is the 95% confidence interval; BMI = body mass index; PASI = psoriasis area and severity index.

Within the psoriasis cases group, the most common type of psoriasis was vulgaris (62%), followed by inverse (31%) and guttate (7%). The distribution of the type of psoriasis between the psoriasis severity subgroups is statistically significantly different (*p* < 0.001), with less cases of inverse psoriasis in the moderate and severe disease subgroups (Table 2). The mean (SD) BMI value was highest among inverse type: 20.9 (±5.56), followed by vulgaris: 18.95 (±5.29) and guttate: 17.06 (±6.5) (*p* = 0.020; Kruskal–Wallis rank sum test). No statistically significant correlation was found between severity, as determined by PASI, and BMI in either vulgaris (*p* = 0.099) or inverse (*p* = 0.9) types (Figure 6). For this analysis, guttate was removed due to its low number of subjects (n = 7).

However, Figure 6 shows that, despite the lack of statistical significance in the correlation between BMI and PASI for the different psoriasis types, the relatively flat slope of the linear regression for inverse (slope = 0.14) is statistically significantly different than the one for vulgaris (slope = 4.38, *p* = 0.0000503), indicating that the BMI values are expected to increase, dependent on the PASI, for the vulgaris but not for the inverse type of psoriasis.

### 3.5. BMI–NAPSI Correlation

A significant proportion of psoriasis patients presented with psoriatic onychopathy (42%), with the most common pattern of nail lesions represented by pitting only (61.9%), followed by pitting + subungual hyperkeratosis (11.9%), pitting + oil drop (9.5%), oil drop only (7.1%), subungual hyperkeratosis only (7.1%), and oil drop + subungual hyperkeratosis (2.5%). The mean (SD) NAPSI value in the subpopulation of psoriasis patients with nail involvement was 8.07 (±3.77), and it increases directly proportional to psoriasis severity (Table 2).

**Figure 7:**
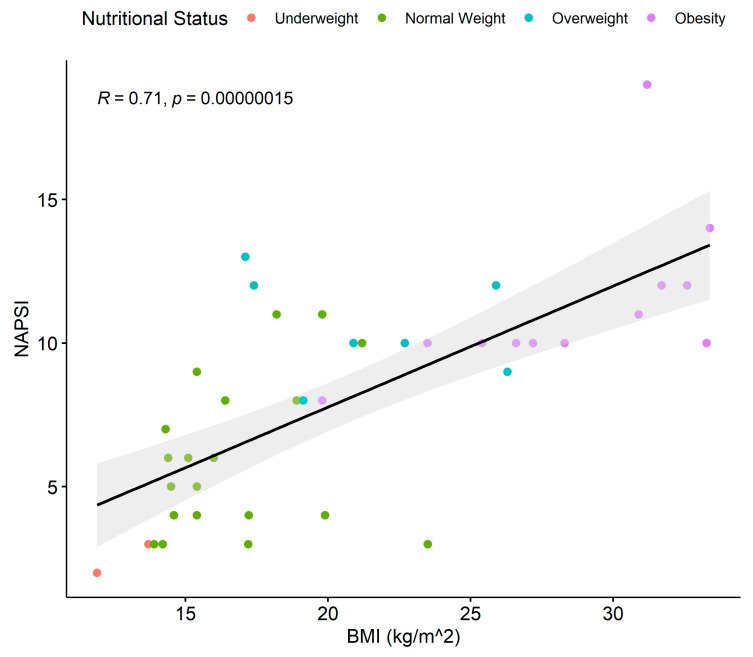
Linear regression between BMI and NAPSI score for psoriasis patients with onychopathy. Dots show the individual data. Colors show the nutritional status of the patients. Black line = regression line; gray area is the 95% confidence interval; BMI = body mass index; NAPSI = nail area and severity index.

Furthermore, in the subgroup of psoriasis patients that presented nail lesions, we identified a statistically significant correlation between the NAPSI score and the BMI following linear regression analysis (*p* = 0.000015) (Figure 7).

## 4. Discussion

In this study, we compared children with various clinical types and severities of psoriasis to age- and sex-matched non-psoriatic controls and identified a statistically significantly increased relative risk of being overweight or obese in psoriasis patients, compared to controls. However, there was no statistically significant correlation between BMI and psoriasis severity (as evaluated by PASI). One potential explanation is that PASI, which is not validated for children, may not accurately reflect the disease severity, due to known differences in morphology and BSA distribution on different anatomical regions between adults and children. Additionally, in our study population, we identified a statistically significant correlation between the severity of nail involvement (as evaluated by NAPSI and BMI), highlighting the fact that BMI increases with the severity of psoriatic onychopathy. In the literature, data support the correlation between the severity of nail involvement and skin lesions and the duration of psoriasis [19,20,21]. Moreover, the features that NAPSI takes into consideration do not change with age. Hence, we can state that, although PASI and BMI did not correlate significantly in our study population, based on the significant association between NAPSI and BMI, the risk of being overweight or obese increases with disease severity, as assessed through nail involvement. Furthermore, in our study we have not found a connection between disease duration and BMI, failing to identify an increasing trend in the BMI with the longer duration of psoriasis. Comparing to some literature data, in which overweight or obese psoriasis patients present with longer disease duration [22], our study presents data from pediatric patients, who, compared to adults, tend to have shorter diseases duration at the time of the analysis. Moreover, the multivariate analysis in our study hinted towards higher BMI values in males, compared to females, without any differences between sexes in terms of BMI and severity. These findings are different to the available literature data on adult psoriasis patients, where no differences were noticed in the sex distribution between different nutritional status groups (i.e., normal weight vs. overweight/obesity) [22]. One potential explanation for the findings in our study could be related to the fact that males, in general, have a higher BMI compared to females, and another one relies on the acknowledged protective role of estrogen in females for metabolic disturbances, including obesity [23]. However, the data analyzed in this study are related to a pediatric population, leaving this topic open for further, additional studies in particular patient population.

In the past decades, the potential correlation between psoriasis and being overweight or obese has been studied more and more, first in the adult population, and later in children. Currently, if, in adults, this association is well-established, with some studies even presenting a direct relationship between obesity and psoriasis severity [24], data in children are scarce and more variable, making it more difficult to establish a relationship across different ages, sexes, and geographical regions. In a Danish cohort study on more than 1000 children aged 7–13 years old, a significant correlation between psoriasis and an increased BMI was found only in girls, strictly at the ages of 12 or 13 years [25]. A case–control study performed in Italy showed that psoriatic children had a considerably higher occurrence of both excessive adiposity (as measured by BMI) and central obesity (assessed through waist-to-height ratio), compared to controls, but only central obesity was independently linked with psoriasis [26]. Another case–control study performed on French patients aged 2–18 years identified a connection only between obesity and psoriasis, and not overweight, with no connection to psoriasis clinical type or severity [27]. Moreover, obesity was found to be a risk factor for psoriasis development in a case–control research conducted in children in the USA [28]. A systematic review of 16 studies identified a substantial rise in the occurrence rates of overweight/obesity in children with psoriasis [6]. An international cross-sectional research performed in 9 countries identified that, on a global scale, regardless of the severity degree, pediatric psoriasis patients have both increased rates of overweight/obesity and central adiposity, compared to controls [9]. However, research performed on Iraqi children identified higher rates of overweight and obesity among children with psoriasis, compared to healthy controls, with a direct connection with psoriasis severity [7]. On the other hand, a prospective cross-sectional case–control study on Australian children with psoriasis (ages 5 to 16 years) failed to identify a significant difference regarding overweight or obesity rates among psoriasis cases compared to healthy controls [8], and another research on children aged 9 to 17 years old with moderate–severe psoriasis failed to identify noteworthy disparities regarding the BMI in BMI patients, compared to controls [29].

There could be multiple explanations for the potential relationship between overweight/obesity and psoriasis; nevertheless, the exact mechanism linking these two medical entities remains unelucidated. The low-grade, chronic, and perpetual inflammatory background is one of the common nominators of both diseases, which leads to a potential bi-directional relationship. Thus, the inflammation present in the adipose tissue in obese patients might contribute to the immune dysregulation seen in psoriasis, which can act both as a promotion and maintenance factor of psoriasis [30]. Psoriasis and obesity share some cytokines with proinflammatory activity, like TNF-α, IL-1, and IL-6 [31] and some adipokines (i.e., adiponectin, leptin, resistin, or lipocalin) with a pathogenic role in both diseases [32,33]. Conversely, psoriasis is linked to metabolic syndrome and insulin resistance, both of which are directly correlated with obesity [34,35]. Gut microbiota seems to be another common pathophysiological key-factor linking psoriasis and obesity, as leaky gut syndrome might be one of the chronic systemic inflammation promotors that contributes to these chronic diseases’ development, and, additionally, gut dysbiosis has been reported in both psoriasis and obesity development [32]. Genetic background represents another common determinant factor between psoriasis and increased BMI, as various gene polymorphisms associated with obesity have been found with potential roles in psoriasis (e.g., PCSK9, FTO, MC4R genes) or the other way around: genetic determinants involved in psoriasis present possible links with obesity (e.g., susceptibility locus HLA-Cw*06, IL21B gene polymorphism) [36]. The effects of weight loss on psoriasis incidence and severity are noteworthy in this potential relationship, as a review of clinical trials showed that diet and weigh loss have a positive effect on psoriasis patients, contributing to PASI score reductions and to a better treatment response, including systemic treatments in such patients [37]. Moreover, it has been shown that bariatric surgery is associated with a lower risk of developing psoriasis [38], and some cases of psoriasis remission after gastric bypass surgery have been reported [39,40].

The strength of this study lies in the physical examination conducted by dermatologists with pediatric expertise, while its limitations include the study design (single center), the small sample study, and a lack of visceral adiposity assessment (through waist/height ratio).

## 5. Conclusions

To the best of our knowledge, this is the first study to evaluate the potential association between pediatric psoriasis and BMI in a cohort of children from Romania across a broad age range. This research highlighted that psoriatic pediatric patients are more likely to be overweight or obese, as compared to controls. Nail involvement is a predictor of psoriasis severity and showed a statistically significant correlation with increased BMI. Further research on a larger sample size and with an improved design (i.e., multi-centric, data collection at multiple visits) is necessary to confirm the association between psoriasis and obesity in children. Nonetheless, screening for overweight/obesity should be considered for pediatric psoriasis patients as a multidisciplinary management of this disease. Early interventions aiming for obesity prevention might help improve psoriasis prognosis and treatment response in such patients.

## Figures and Tables

**Table 1:** Summary of the study population’s characteristics by cases and control groups.

	Controls (Non-Psoriasis) (N = 100)	Cases (Psoriasis) (N = 100)	*p*-Value *
Age (years)			0.99
Mean (SD) [CV%]	10.2 (4.43) [43.5%]	10.2 (4.43) [43.5%]	
Median [Min, Max]	10.0 [1.00, 17.0]	10.0 [1.00, 17.0]	
Weight (kg)			0.57
Mean (SD) [CV%]	38.7 (19.2) [49.5%]	41.9 (23.2) [55.3%]	
Median [Min, Max]	31.4 [10.5, 100]	33.5 [8.50, 106]	
Sex			0.99
Female	47 (47.0%)	47 (47.0%)	
Male	53 (53.0%)	53 (53.0%)	
BMI (kg/m^2^)			0.68
Mean (SD) [CV%]	18.4 (3.95) [21.4%]	19.4 (5.52) [28.4%]	
Median [Min, Max]	17.7 [13.7, 40.1]	17.4 [11.9, 34.7]	
Nutritional Status			<0.001
Underweight	0 (0%)	5 (5.0%)	
Normal Weight	95 (95.0%)	64 (64.0%)	
Overweight	3 (3.0%)	14 (14.0%)	
Obesity	2 (2.0%)	17 (17.0%)	
Age Group			>0.99
Toddler (1 ≤ 2 years)	1 (1.0%)	1 (1.0%)	
Early childhood (2 ≤ 6 years)	18 (18.0%)	18 (18.0%)	
Middle childhood (6 ≤ 12 years)	41 (41.0%)	41 (41.0%)	
Adolescents (12–17 years)	40 (40.0%)	40 (40.0%)	
Nail involvement			NA
No	100 (100%)	58 (58.0%)	
Yes	0 (0%)	42 (42.0%)	

SD—standard deviation, CV%—coefficient of variance, min—minimum, max—maximum. *: Wilcoxon rank sum test for continuous variables; Pearson’s chi-squared test and Fisher’s exact test for categorical variables.

**Table 2:** Cases (psoriasis) group characteristics.

	Mild (N = 65)	Moderate (N = 25)	Severe (N = 10)	*p*-Value *
Age (years)				0.41
Mean (SD) [CV%]	10.4 (4.37) [42.1%]	9.28 (4.34) [46.7%]	11.2 (5.22) [46.6%]	
Median [Min, Max]	10.0 [1.00, 17.0]	8.00 [4.00, 17.0]	13.5 [3.00, 17.0]	
Weight (kg)				0.19
Mean (SD) [CV%]	41.5 (22.2) [53.5%]	38.1 (22.8) [59.9%]	53.6 (28.4) [52.9%]	
Median [Min, Max]	38.0 [8.50, 105]	25.0 [16.0, 84.0]	53.4 [21.6, 106]	
Sex				0.18
Female	35 (53.8%)	9 (36.0%)	3 (30.0%)	
Male	30 (46.2%)	16 (64.0%)	7 (70.0%)	
BMI (kg/m^2^)				0.25
Mean (SD) [CV%]	19.3 (5.03) [26.1%]	19.0 (6.38) [33.5%]	21.6 (6.35) [29.4%]	
Median [Min, Max]	18.1 [12.8, 34.7]	16.3 [11.9, 33.4]	21.9 [13.9, 31.7]	
Nutritional Status				0.79
Underweight	3 (4.6%)	2 (8.0%)	0 (0%)	
Normal Weight	43 (66.2%)	15 (60.0%)	6 (60.0%)	
Overweight	8 (12.3%)	5 (20.0%)	1 (10.0%)	
Obesity	11 (16.9%)	3 (12.0%)	3 (30.0%)	
Age Group				0.11
Toddler (1 -< 2 years)	1 (1.5%)	0 (0%)	0 (0%)	
Early childhood (2 -< 6 years)	8 (12.3%)	7 (28.0%)	3 (30.0%)	
Middle childhood (6 -< 12 years)	29 (44.6%)	11 (44.0%)	1 (10.0%)	
Adolescents (12–17 years)	27 (41.5%)	7 (28.0%)	6 (60.0%)	
Nail involvement				0.27
No	41 (63.1%)	11 (44.0%)	6 (60.0%)	
Yes	24 (36.9%)	14 (56.0%)	4 (40.0%)	
PASI Score				<0.001
Mean (SD) [CV%]	2.18 (1.17) [53.4%]	6.89 (1.50) [21.8%]	16.1 (7.64) [47.6%]	
Median [Min, Max]	2.00 [0.300, 4.80]	6.80 [5.00, 9.80]	13.4 [10.4, 35.2]	
NAPSI				0.40
Mean (SD) [CV%]	2.82 (4.17) [148%]	3.56 (4.55) [128%]	4.50 (6.93) [154%]	
Median [Min, Max]	0 [0, 13.0]	2.00 [0, 14.0]	0 [0, 19.0]	
CDLQI Score				<0.001
Mean (SD) [CV%]	5.35 (5.21) [97.4%]	9.28 (7.06) [76.1%]	12.2 (5.35) [43.9%]	
Median [Min, Max]	4.00 [1.00, 25.0]	8.00 [1.00, 28.0]	13.5 [5.00, 20.0]	

* Kruskal—Wallis rank sum test; Fisher’s exact test.

## Data Availability

The original contributions presented in the study are included in the article/Supplementary Material, further inquiries can be directed to the corresponding author.

## Supplementary Materials

The following supporting information can be downloaded at: https://www.mdpi.com/article/10.3390/children11070885/s1, Figure S1: A pairwise comparison of multivariate data focusing on the differences between sexes.
