# Supplementary material for: Nutritional Status in Pediatric Psoriasis: A Case–Control Study in a Tertiary Care Referral Centre
Source: Children (Basel). 2024 Jul 22;11(7):885. doi: 10.3390/children11070885 (PMC11275588; doi:10.3390/children11070885)
Supplement: Supplementary file 1 [file children-11-00885-s001.zip › children-3054059-supplementary.pdf]

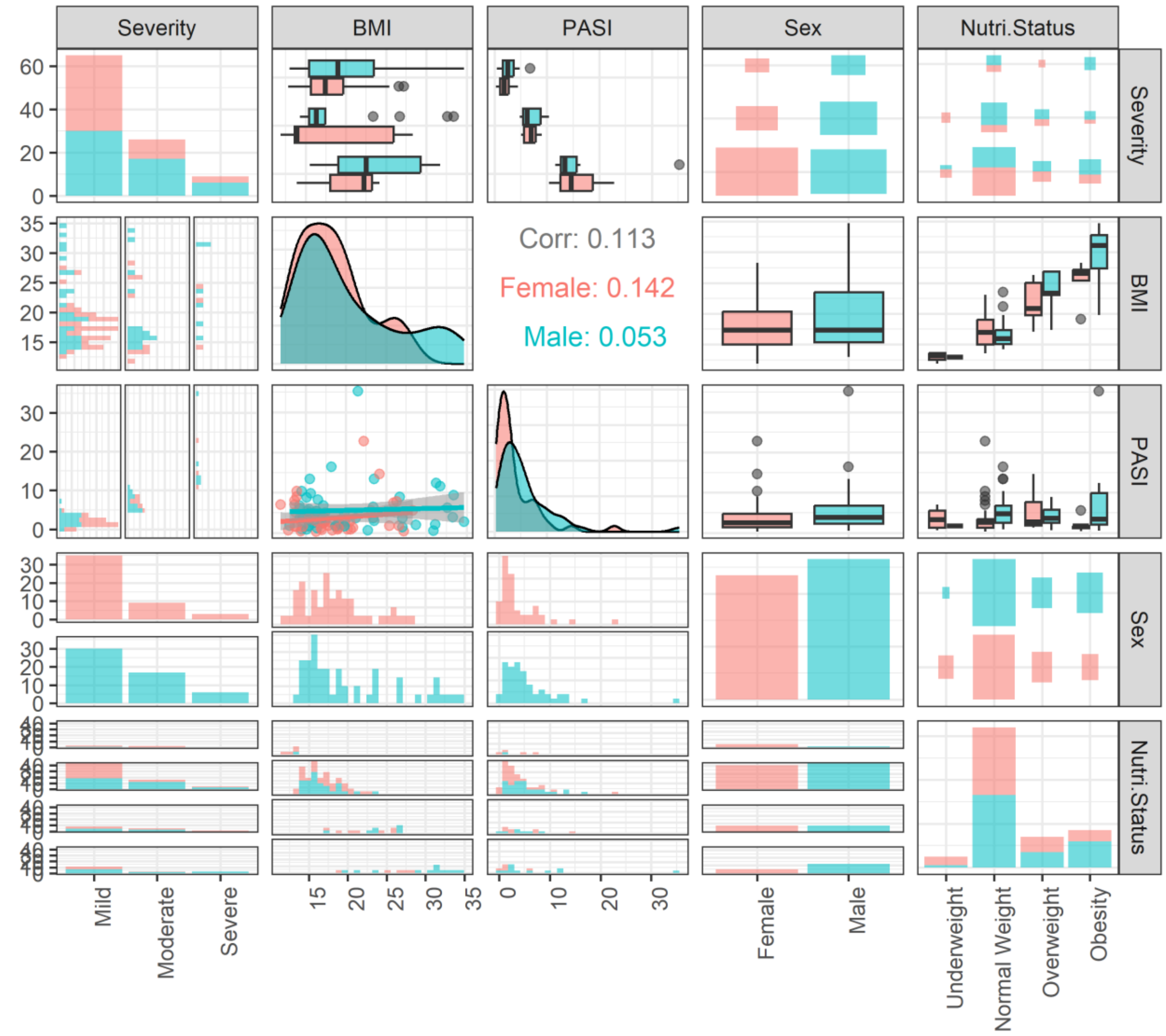

Figure S1 - A pairwise comparison of multivariate data focusing on the differences between sexes – males in blue and females in pink. This provides two different comparisons of each pair of columns and displays either the density or count of the respective variable along the diagonal.
